# Adult Lifespan Cognitive Variability in the Cross-Sectional Cam-CAN Cohort

**DOI:** 10.3390/ijerph121215003

**Published:** 2015-12-07

**Authors:** Emma Green, Meredith A. Shafto, Fiona E. Matthews, Cam-CAN, Simon R. White

**Affiliations:** 1 Department of Public Health and Primary Care, Cambridge Institute of Public Health, Univeristy of Cambridge, Cambridge CB2-0SR, UK; ep382@medschl.cam.ac.uk; 2 Centre for Speech, Language and the Brain, Department of Psychology, University of Cambridge, Downing Street, Cambridge CB2-3EB, UK; mshafto@csl.psychol.cam.ac.uk; 3 MRC Biostatistics Unit, Cambridge CB2-0SR, UK; fiona.matthews@mrc-bsu.cam.ac.uk; 4 Institute of Health and Society, Newcastle University, Newcastle NE4-5PL, UK; 5 Cambridge Centre for Ageing and Neuroscience (Cam-CAN), University of Cambridge and MRC Cognition and Brain Sciences Unit, Cambridge, UK, www.cam-can.com; ccmanagement@cam-can.com

**Keywords:** cognitive variability, adult lifespan, heterogeneity, MMSE, ceiling effects, variance confounders, verbal fluency, episodic memory

## Abstract

This study examines variability across the age span in cognitive performance in a cross-sectional, population-based, adult lifespan cohort from the Cambridge Centre for Ageing and Neuroscience (Cam-CAN) study (*n* = 2680). A key question we highlight is whether using measures that are designed to detect age-related cognitive pathology may not be sensitive to, or reflective of, individual variability among younger adults. We present three issues that contribute to the debate for and against age-related increases in variability. Firstly, the need to formally define measures of central tendency and measures of variability. Secondly, in addition to the commonly addressed location-confounding (adjusting for covariates) there may exist changes in measures of variability due to confounder sub-groups. Finally, that increases in spread may be a result of floor or ceiling effects; where the measure is not sensitive enough at all ages. From the Cam-CAN study, a large population-based dataset, we demonstrate the existence of variability-confounding for the immediate episodic memory task; and show that increasing variance with age in our general cognitive measures is driven by a ceiling effect in younger age groups.

## 1. Introduction

Age-related changes in cognition can be informed by both changes in mean performance, a measure of central tendency, and changes in variance, a measure of variability, within and between subjects. Whereas declines in mean performance with age are in many cases expected, researchers have been interested in age-related changes in variance as another way to understand changes in cognition.

Cross-sectional studies of normal ageing typically find age-related declines in a range of cognitive functions, including memory, attention, processing speed and executive function (see [1,2,3] for recent reviews). In addition to declines in mean performance with age, it is often claimed that groups of older adults have higher inter-subject variability than younger adults [4,5,6,7,8,9]. We assert that some of the reported increases in variability in older adults can be explained by non-representative samples and difficulties with the statistical properties of the scores. Age-related increases in heterogeneity, if true, may reflect mechanisms that are important for understanding cognitive ageing, including the accumulation of sources of individual differences over the adult lifespan, or age-related increases in intra-individual variability due to deterioration of cognitive functions (e.g., [10]). Morse [11] conducted a meta-analysis of studies on response time, memory, and intelligence and found that older groups were more variable in measures of response time, memory, and fluid intelligence, but not for measures of crystallised intelligence. Shammi *et al.* [12] found evidence for age-related increases in inter-subject variability in both psychomotor (e.g., choice RT) and higher level cognitive functions, but also found that age differences were sensitive to specific task demands. More recent studies provide similar evidence that while some measures show age-related increases in inter-subject variability, others do not [5,13].

The mixed results from these studies may be due to a number of factors, including the use of volunteer samples which are likely to be non-representative [11] and favour higher homogeneity within younger groups who are often drawn from student populations or from populations with above average educational attainment. Moreover, age-related increases in variance may reflect biases in the nature of the tasks that are examined. Performance measures under examination in ageing research tend to show age-related declines in mean performance (e.g., intelligence or episodic memory; [11,13]). This likely reflects the focus of ageing research on understanding age-related cognitive decline in domains that are affected in pathological ageing including dementia.

The current study examines variance across the age span in cognitive performance in a cross-sectional, population-based, adult lifespan cohort from the Cambridge Centre for Ageing and Neuroscience (Cam-CAN) study. A key question we highlight is whether using measures that are designed to detect age-related cognitive pathology may not be sensitive to, or reflective of, individual variance among younger adults.

Using the Cam-CAN study we consider six cognitive measures, as representative examples of commonly used measures in cognitive studies, and measures that are claimed to exhibit age-related increases in variability. We begin Section 2 by giving details of the study and our measures of interest.

In this paper we discuss three issues that contribute to the debate for and against age-related increases in variability. Firstly that the term “variability” is not strictly defined, having a colloquial usage. In Section 2.2 we explicitly define measures of central tendency, also known as measures of location, and measures of variability, also known as measures of spread. Henceforth we reserve the term variability to cover all forms of changing measures over the lifespan, and take care to specify changing measures of location or spread as appropriate. Secondly, that increases in measures of spread (e.g., variance) may actually be due to confounder sub-groups with stable spread but shifting location; in Section 3.3 we illustrate this concept with simulated data and demonstrate the existence of this effect for the immediate episodic memory. Finally, that increases in spread may be a result of ceiling effects at earlier ages; where the measure is not sensitive enough and a large number of individuals attain the maximum score. In Section 3.4 we discuss the problem of comparing variance across the adult lifespan in the presence of a ceiling effect for both our general cognitive measures.

## 2. Methods

### 2.1. Cam-CAN Study

As recent years have observed dramatic increases in average life expectancy, there is an ever-growing need to understand the factors involved in ageing healthily. The Cam-CAN study was developed to explore the underpinnings of successful cognitive ageing. The data is a rare and valuable resource as not only does it allow changes to be explored across the adult lifespan from age 18 upwards, but as individuals were recruited randomly from the general population via primary care population lists, the study has the advantage of being as close as possible to a representative sample of the population. This provides a unique opportunity to identify factors associated with successful ageing in a non-volunteer cohort.

The study design involved testing participants across three stages (see Shafto *et al.* [14] for further details). Stage 1 recruited 2680 individuals with background measures such as age, gender and education attainment being gathered as part of a questionnaire administered to participants within their own homes by trained interviewers. The cognitive measures reported here were gathered as part of a wider cognitive battery [14]. To attain a measure of general cognitive status, participants undertook the Mini-Mental State Examination (MMSE; [15]), and the Addenbrooke’s Cognitive Examination Revised (ACE-R; [16]). Episodic memory was assessed using measures of immediate and delayed story recall from the logical memory sub-test of the Weschler Memory Scale Third UK edition (WMS-III UK; [17]). Two measures of verbal fluency were also recorded: category fluency as the number of unique animals produced in one minute; and letter fluency as the number of unique words beginning with the letter P (not names of people or places) in one minute. The measures presented within this paper were given to all individuals across the adult age range and were chosen to demonstrate measures with continuous distributions and truncation.

Participants were asked their educational background, from which their education attainment (highest qualification) was derived and coded as: None, GCSE/O-level, A-level, or Degree (see [14] for details). Table 1 gives the participant counts by age-group, and subdivided by sex-group and education-group.

**Table 1:** Cam-CAN first-stage participants by age-group, sex, and educational attainment. Individuals were a random sample from the complete population until the end of the study accrual period.

Age	Sex		Highest Qualification					Total
	Female	Male	None	GCSE	A-Level	Degree	Unknown	
18–27	117	87	3	38	57	106	0	204
28–37	207	157	5	34	47	277	1	364
38–47	194	153	7	44	57	239	0	347
48–57	131	120	11	34	56	150	0	251
58–67	199	160	48	55	73	182	1	359
68–77	191	207	90	58	78	168	4	398
78–87	374	246	193	73	153	188	13	620
88+	95	42	55	12	32	31	7	137
Total	1508	1172	412	348	553	1341	26	2680

Ethical approval for the study was obtained from the Cambridgeshire 2 (now East of England-Cambridge Central) Research Ethics Committee. Participants gave written informed consent.

### 2.2. Variability in Terms of Statistical Properties: Measures of Location and Measures of Spread

For a univariate outcome (e.g., total MMSE score) measured on a complete population there will typically be a range of scores, and for clinically-relevant measures like the MMSE the range must be reasonable, interpretable, and clinically-relevant for the measure to have utility. The collection of the individual scores (the sample) will exhibit a distribution that should reflect the range of scores seen in the complete population; it may follow one of the standard statistical distributions, e.g., normal, binomial, Poisson, and may be continuous, discrete (counts,ordinal), or categorical.

Our question of interest is what the sample tells us about the variability of the score across the adult lifespan, that is, how does the distribution of scores change over the age-groups and how do different samples impact on the conclusions of variability?

The sample can be summarised in several ways, which can be generally grouped into two classes: measures of location and measures of spread. The mean and median are two common measures of location, representing in some sense a “typical” score. The inter-quartile-range, range, and variance are common measures of spread, giving some information as to how similar or varied the set of scores are. Note that measures of spread may be defined with respect to a measure of location. For example, the variance is a measure of spread with respect to the mean.

### 2.3. Statistical Multiple Group Tests for Median and Variance

In order to test for differences between age-groups we used statistical tests that did not make a priori assumptions about distributions.

Many standard statistical tests such as the t-test for equality of means and the F-test for equality of variances are so-called parametric tests. Their validity depends on a set of assumptions, typically about the distributional form. In our setting, where we wish to investigate changes in location and spread, we cannot a priori make these assumptions.

As an alternative we consider non-parametric statistical tests, these generally have weaker (distributional) assumptions (often based on ranks or order statistics). However, there is a cost to the increased flexibility from the weaker assumptions, typically these tests have a lower statistical power to detect a true effect compared to a properly specified parametric test of the same hypothesis.

In order to test whether all age-groups have an equal median we use Mood’s median test [18]. Similarly, to test whether all age-groups have an equal variance we use Fligner’s test for equal variance [19]. Both tests are non-parametric and make no assumptions about the shape of the distribution. Importantly, Mood’s test does not require equality of spread, and Fligner’s test does not require equality of location.

### 2.4. Illustrating Variability: The Box Plot

In order to visualise the spread of performance scores across age-groups, we used box plots to convey multiple measures of location and spread across sub-groups.

It is difficult to convey sample distributions, especially across multiple sub-groups, with a view to capturing the important aspects. A common illustration for a single sample would be a histogram or (kernel) density plot, which conveys the location and spread of the scores. However, for comparing many sub-groups the plots quickly become cluttered and difficult to assess.

Conversely, we can simply report single-number summaries by sub-group in a table, e.g., report the mean and variance (or standard deviation) within each sub-group. However, reducing from a distribution to a single-number, or pair of numbers, can drastically misrepresent the original distributions. Summary statistics are, by their definition, a low dimensional summary of a more complex object, in this case the population/sub-group distribution.

The box plot represents a compromise, being a coarser representation of the data than a histogram, but conveying multiple measures of location and spread across sub-groups in a reasonably concise way. Within each sub-group a box plot depicts: the median and inter-quartile-range as the “box”, the size of the sub-group as the “box-width” (proportional to square-root of the sub-group size, *i.e.*, $\sqrt{n_{i}}$), the “whiskers” extend to the smallest/largest data value that is greater/less than 1.5 × IQR below/above the lower/upper quartile, and any outliers (*i.e.*, data values beyond the whiskers) as outlier-circles. Note, the definition of a box plot can vary, in particular the definition of the “whiskers” and “outlier” may differ.

### 2.5. Truncation: Ceiling and Floor Effects

The range of some measures may not be appropriate across the adult lifespan, in particular the MMSE as a measure of pathological ageing including dementia, was not necessarily designed to be given to young adults. That is, the MMSE has little discriminatory power for high-functioning individuals because they may all score near the top of scale (the maximum MMSE score is 30).

This is a so-called ceiling effect. Implicitly we are saying that the MMSE would need a wider range of questions to be able to discriminate among the healthy respondents (such as the Modified MMSE [20]). Thus we hypothesise that there exists a larger set of values for the MMSE that could discriminate among those individuals who are affected by the ceiling effect. Floor effects are the opposite case, when individuals are at the bottom of the scale.

If there is a ceiling effect on the MMSE score then this will impact our measures of location and spread; as these measures will reflect the truncated scores, not necessarily the “true” spread of abilities. The degree to which the truncation causes the measure of spread to be a poor reflection of the “true” spread directly impacts the interpretability of the measure.

We are interested in characterising the changing score distribution across the age-groups. If the ceiling effect is consistent across the age-groups then the impact on the distributions would also be consistent, implying tests comparing the distributions would be valid. However, if the ceiling effect varies then we must take care to interpret any tests.

### 2.6. Simulated Data as Illustration

We use simulated data to illustrate some of the issues of interpreting output from statistical tests and box plots. To help compare and interpret the Cam-CAN box plots, and measures of location and spread, we generated the simulated dataset based on the Cam-CAN measure of category fluency; with known properties we hope to recover in the tests and plots.

Also following the Cam-CAN data, we generated the simulated dataset (*n* = 2400) with age-groups and education-groups such that *n_ij_* = 150 for *i* = {18−27}, ⋯, {88+} and *j* = {≤GCSE}, {≥ A-level}. Each individual, *l*, within an age-education-group (*l* = 1, ⋯, *n_ij_*) has a score, *x*, generated according to a negative binomial, *i.e.*, *x_ijl_* ∼ NegBin (mean_*ij*_, sd_*ij*_).

We specify the baseline mean and standard deviation, mean = 30 and sd = 5.5. We can keep these fixed across all sub-groups or vary by adding sub-group effects. Specifically, we add a constant step for each age-group; the mean decreased in steps of 1.5, and the variance increased in steps of 1 (*i.e.*, for the 88+ age-group the mean would be, 30 − 7(1.5) = 30 − 10.5 = 19.5, and the standard deviation would be, 5.5 + 7(1) = 5.5 + 7 = 12.5). Scenario A is defined to have a fixed variance and decreasing mean, Scenario B is defined to have a increasing variance and fixed mean, and Scenario C is defined to have increasing variance and decreasing mean.

Simulated Scenario D uses the education-group to define a mixture score. We specified the mean_*ij*_ to be fixed for *j* = {≥ A-level} and varying for *j* = {≤GCSE}; the age-education-group variance was held fixed.

## 3. Results and Discussion

In the following sections we consider evidence of variability across the adult lifespan using the Cam-CAN study dataset and our simulated datasets. Continuing our main theme of explaining whether there is evidence of variability, having discussed the concept in Section 2.2, we now use box plots and statistical tests to investigate our datasets. Section 3.1 considers simple measures of changing location and spread in the Cam-CAN dataset, where we discover very different looking box plots for our six cognitive scores (two memory, two verbal fluency, two general cognition).

In Section 3.2 we illustrate box plots and statistical tests for our simulated dataset, thus we have a known truth with which to assist our interpretation of the output. However, we have difficulty relating our simulated Scenario D to the known truth, which leads us to Section 3.3 where we consider the impact of confounders on measures of spread. We discover that our results on the memory scores from Section 3.1 are altered by considering sex and education sub-groups.

Finally, in Section 3.4 we consider the issue of truncation, specifically ceiling effects, on the general cognition scores (MMSE and ACE-R), comparing with our simulated truncation.

When considering statistical significance the commonly accepted “standard” is a *p*-value threshold of 5%, this is a reasonable level of evidence against the null hypothesis (note for both Mood and Fligner’s tests, the null hypothesis is that all groups have equal median or variance respectively). However, we are performing a slightly exploratory analysis in this paper, which might lead us to consider adjusting our required level of evidence due to some issues of multiple testing; without going so far as to apply formal adjustments, e.g., Bonferroni corrections [21]. Further, the Cam-CAN dataset is very large (*n* = 2680) compared to many cognitive studies, so we can perhaps view our evidence as needing to be slightly stricter. For these reasons, in the following sections we consider evidence for or against the null hypothesis rather than a strict significant or non-significant statement; although if we were to speak in terms of the latter we would be considering a 1% threshold as evidence against the null, and less than 0.1% as strong evidence.

The simulated dataset, plots and analyses were generated using GNU R v3.2.0 [22].

### 3.1. Age and Heterogeneity: Challenging Perceived Wisdom about Ageing and Variability

A common belief in ageing research is that variability increases with age; that is commonly meant to imply that the variance increases with age, for many cognitive traits [4,5,6,7,8,9].

**Figure 1:**
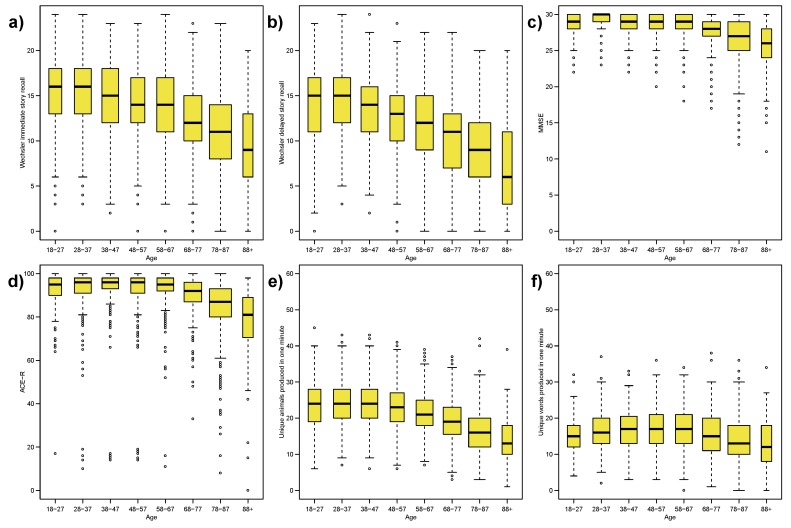
Box plots of Cam-CAN data by age-groups: (**a**) Wechsler immediate story recall; (**b**) Wechsler delayed story recall; (**c**) Mini-Mental State Examination; (**d**) Addenbrooke’s Cognitive Examination Revised; (**e**) category fluency; (**f**) letter fluency. Note that the verbal fluency measures are not within a bounded range, unlike the MMSE (0–30) or story recall (0–24); the plot ranges from 0–60 to reflect a limit of one response per second. Definition of box plot: box indicates the inter-quartile range, mid-line indicates the median, box-width proportional to square-root of sample size (*i.e.*, $\sqrt{n_{i}}$), whiskers extend to largest data value within 1.5 × IQR of the respective quartile, circles correspond to outliers (*i.e.*, data values beyond the whiskers).

Figure 1a,b show box plots of the immediate and delayed memory performance within age-groups, which provides evidence of worse performance in older adults. However the box-height, representing the inter-quartile-range, and whisker-length, representing the range, appears to remain consistent - primarily on a visual inspection - across the age-groups. These plots seem to present counter examples to the idea that there is always increasing variance in cognitive measures with age.

To formally check our visual impressions we use Mood’s and Fligner’s tests. Table 2 gives the *p*-values from these tests, Mood’s median test confirms our impression of a decline across the age-groups with strong evidence against a constant median. Fligner’s test confirms our impression of constant variance for the Wechsler delayed story recall score, with no evidence against the null hypothesis of equal variance across the age-groups. However, Fligner’s test gives strong evidence against constant variance for the Wechsler immediate story recall score, counter to our visual impression.

**Table 2:** Results of non-parametric statistical tests for equality of median and variance across all groups for measures in Figure 1. For clarity, *p*-values less than 0.001 are reported as *<0.001*.

Figure	Outcome	Mood’s Median Test	Fligner’s Variance Test
1a	Wechsler immediate story recall	*<0.001*	0.004
1b	Wechsler delayed story recall	*<0.001*	0.121
1c	MMSE	*<0.001*	*<0.001*
1d	ACE-R	*<0.001*	*<0.001*
1e	Category fluency	*<0.001*	0.013
1f	Letter fluency	*<0.001*	0.018

Despite the strong visual similarity between Figure 1a,b, the formal statistical test indicates changes in spread across the adult lifespan. Recall that Mood’s and Fligner’s tests have an alternative hypothesis that at least one sub-group is different. There is no indication whether it is one or more groups, nor which specific groups are different.

In Figure 1e,f we compare category fluency and letter fluency, as measures of verbal fluency. For letter fluency the location appears visually stable across all age-groups, but Mood’s test gives strong evidence against equal medians. On visual inspection, Figure 1e,f show a relatively stable variance. Fligner’s variance test indicates some evidence against equal variances (as discussed earlier, at the “traditional” 5%-level, but not at a 1%-level).

Considering the stronger evidence threshold of 1% (larger dataset and multiple testing), we can say that delayed memory recall and verbal fluency have little evidence of age differences in variance, despite age-related declines in performance.

### 3.2. Simulated Data: What Do True Effects Look Like?

As illustrated in Section 3.1, our visual impressions can be misleading and inconsistent with a formal statistical test. To help interpret box plots, and to illustrate some potential pitfalls, we produced similar plots to those in Section 3.1 using our simulated dataset. Hence there is a known truth to compare to.

**Figure 2:**
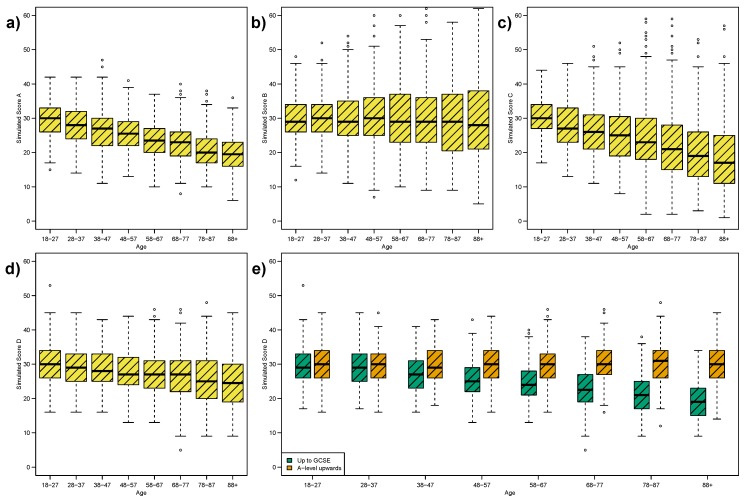
Box plots of simulated data by age-groups: (**a**) declining mean and fixed variance; (**b**) fixed mean and increasing variance; (**c**) declining mean and increasing variance; (**d**) Simulated Scenario D is a mixture of two sub-groups (≤ GCSE, ≥A-level) with declining mean and fixed mean respectively, and both with fixed variance; as illustrated in (**e**). As a result of this mixture of distributions, the combined Scenario D appears similar to Scenario C; within (**d**) there is an unmeasured confounder.

Figure 2a–c illustrate the box plots for generated Scenarios A, B and C respectively, when the true median and variance are either fixed or varying. The simulated data are based on category fluency (see Figure 1e). Table 3 gives the corresponding Mood and Fligner tests alongside the “truth” in each case. We see that Mood and Fligner’s tests give *p*-values as expected in each case.

**Table 3:** Results of non-parametric statistical tests for equality of median and variance across all groups for measures in Figure 2. The “Truth” column indicates whether the median or variance are fixed or varying (declining and increasing respectively) across the age-groups.

Figure	Outcome	Median		Variance	
		Truth	Mood’s Test	Truth	Fligner’s Test
2a	Scenario A	Varies	*<0.001*	Fixed	0.779
2b	Scenario B	Fixed	0.179	Varies	*<0.001*
2c	Scenario C	Varies	*<0.001*	Varies	*<0.001*
2d	Scenario D	Mixture	*<0.001*	Fixed	*<0.001*
2e	Scenario D, ≤GCSE	Varies	*<0.001*	Fixed	0.909
2e	Scenario D, ≥A-Level	Fixed	0.884	Fixed	0.592
5a	Scenario A, Truncated	Varies	*<0.001*	Fixed	0.001

Figure 2d for Scenario D looks very similar to Figure 2c for Scenario C, with similar statistical test values (see Table 3). Thus our visual impression is confirmed by the formal test. However, Scenario D is actually a mixture of fixed and varying medians. As described in Section 2.6, we have generated two sub-groups based on education attainment (≤GCSE, ≥A-level) with declining and fixed medians respectively. The sub-groups are illustrated separately in Figure 2e.

This is an illustration of the fallacy of an unmeasured confounder, from Scenario D we would conclude strong evidence against fixed variance. However, when we inspect Scenario D by sub-group we see the truth reflected as no evidence against fixed variance, see Table 3.

### 3.3. Confounders and Variability

Although researchers are used to considering the role of confounds on mean performance (*i.e.*, nuisance factors and potential cohort differences), these confounders can also impact variance as illustrated by our simulated data in Section 3.2.

As illustrated in Figure 2d,e, a key weakness of the box plot in depicting a distribution is the failure to illustrate a multi-modal or mixture distribution. That is, if the distribution of outcome scores is a mixture of several distinct sub-groups, then in that case, the distribution within the sub-groups may be similar in location or spread, but when viewed as a combined set may be different in location and different in spread.

With confounders in mind, we revisit the two memory recall tests as they have the no evidence and the strongest evidence against equal variances. Firstly the delayed story recall score which showed declining median and constant variance, and secondly the immediate story recall score which showed declining median and varying variance.

**Figure 3:**
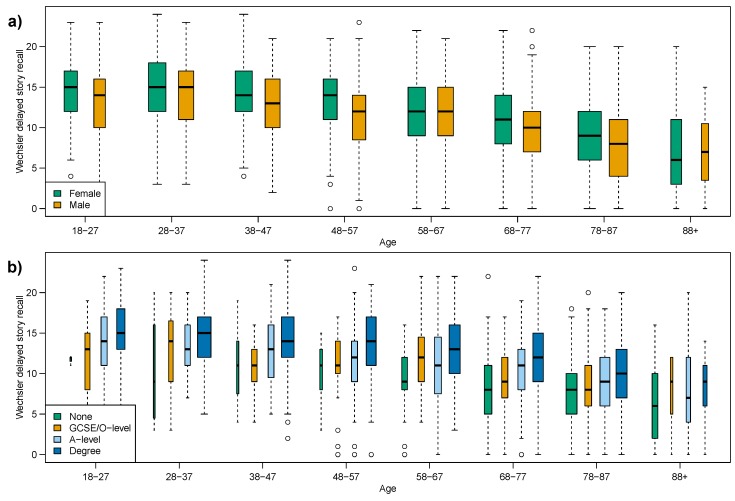
Box plots of delayed story recall, subdividing age-groups by: (**a**) sex; (**b**) education attainment.

The delayed story recall (Figure 1b), which showed no evidence against constant variance, is subdivided in Figure 3a by sex and in Figure 3b by education. At this point it is important to note that the sample size within some of these sub-groups is substantially smaller than others. Thus there is an increase in sampling-error in our comparisons. Table 4a indicates that when considering the sex and education sub-groups there is a weakening of the evidence against equal variance for Females and those with a Degree equivalent education. However the overall conclusion remains the same, there is no evidence against a constant variance.

**Table 4:** Results of non-parametric statistical tests for equality of median and variance across all age-groups for story recall, by sex and by education attainment, corresponding to Figure 3 and Figure 4 for the delayed and immediate tests respectively.

(a) Wechsler Delayed Story Recall				(b) Wechsler Immediate Story Recall			
Figure	Sub-Group	Mood’s Median Test	Fligner’s Variance Test	Figure	Sub-Group	Mood’s Median Test	Fligner’s Variance Test
1b	*All*	*<0.001*	0.121	1a	*All*	*<0.001*	0.004
3a	Female	*<0.001*	0.026	4a	Female	*<0.001*	0.002
3a	Male	*<0.001*	0.572	4a	Male	*<0.001*	0.561
3b	None	*<0.001*	0.054	4b	None	0.010	0.155
3b	GCSE	*<0.001*	0.338	4b	GCSE	0.016	0.355
3b	A-level	*<0.001*	0.292	4b	A-level	*<0.001*	0.441
3b	Degree	*<0.001*	0.026	4b	Degree	*<0.001*	0.121

The effect of considering sub-groups is far greater on the immediate story recall score, which showed strong evidence against a constant median and strong evidence against a constant variance. Table 4b shows that when considering education-groups, Fligner’s test indicates a reversal of outcome such that there is no evidence against equal variance across the adult lifespan; and the characteristically strong evidence against equal medians in Mood’s test gives way to weaker evidence for the None and GCSE/O-level education-groups.

**Figure 4:**
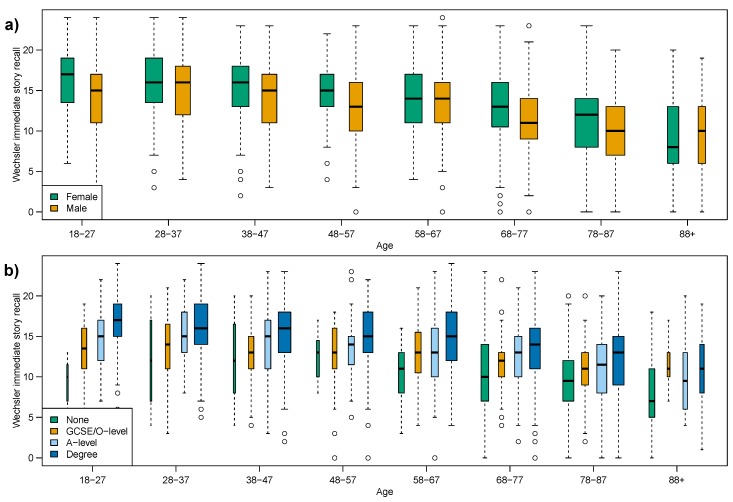
Box plots of immediate story recall, subdividing age-groups by: (**a**) sex; (**b**) education attainment.

When considering immediate story recall score by education-groups we conclude no age-related change in variance and further, for the first measure in this paper, no evidence (at 1% level) of a changing median in the None and GCSE/O-level sub-groups.

The effect of considering sex sub-groups is not as dramatic as the education-groups, but we see no evidence against equal variances for Males.

### 3.4. Interpreting Variability in the Presence of Truncation: Floor Effects and Ceiling Effects

Inspecting Figure 1c,d we see strong indicators that the MMSE, and to a lesser extent the ACE-R, measures might suffer from a ceiling effect.

**Figure 5:**
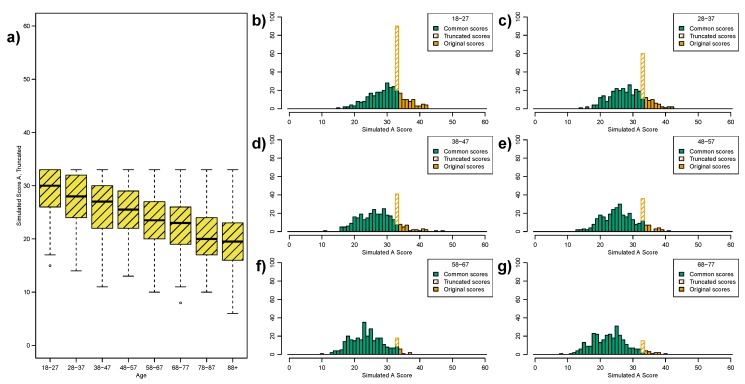
Illustration of the effect of truncating the range of simulated Scenario A to [0,33] (original scale was unbounded, *i.e.*, [0,∞]). (**a**) Box plot of truncated score, contrast with original in Figure 2a; (**b**–**g**) Comparisons of the histograms for the truncated and original scores over the first six age-groups. All original scores above 33 are truncated, *i.e.*, recoded, as 33. Hence the large spikes in the truncated score histograms.

To illustrate the impact a ceiling effect might have on variance across age groups we return to our simulated dataset, specifically Scenario A (Figure 2a). We generate a truncated version of Scenario A, such that any value greater than 33 is recoded as 33. Figure 5a shows the resulting box plot, for direct comparison with Figure 2a, we see a similar box plot pattern as in the MMSE.

Figure 5b–g compare the histograms for the original and truncated scores across the first six age-groups. The clear feature present in the early histograms is a significant spike at the truncation point.

Ceiling effects will impact on measures of location and spread differently. Table 5 compares the mean, median and standard deviation for the original and truncated Scenario A. The mean is, as expected, underestimated whereas the median is more robust. However, the variance is significantly biased. Recall that the variance is defined in terms of the mean, thus the truncation is having a double impact on the estimated variance.

**Table 5:** Comparison of the mean, median and standard deviation across all age-intervals for the original and truncated simulated Scenario A. The true distribution of simulated Scenario A has a declining mean (and median) with constant variance. The median is robust to truncation, as long as the true median is less than the truncated score. The mean is, as expected, under-estimated in the lower age-groups; although in this example the effect is quite small. The variance (standard deviation) is incorrectly estimated for the truncated score.

Age	Mean		Median		Standard Deviation	
	All	Truncated	All	Truncated	All	Truncated
18–27	29.84	28.96	30.00	30.00	5.31	4.13
28–37	28.11	27.57	28.00	28.00	5.28	4.44
38–47	26.79	26.32	27.00	27.00	5.68	4.80
48–57	25.71	25.47	25.50	25.50	5.27	4.81
58–67	23.80	23.74	23.50	23.50	4.99	4.88
68–77	22.95	22.82	23.00	23.00	5.48	5.19
78–87	20.51	20.47	20.00	20.00	5.33	5.22
88+	19.59	19.56	19.50	19.50	5.52	5.44

Comparing our simulated Scenario A to Scenario D, where the former represents the “true” ability of individuals, we would conclude from Figure 5a that the variance in Scenario D increases across the adult lifespan; this is confirmed by Fligner’s test in Table 3. However, we know that the variance is in fact constant across the adult lifespan. Hence, the ceiling effect results in a false representation of the variance as a measure of spread.

**Figure 6:**
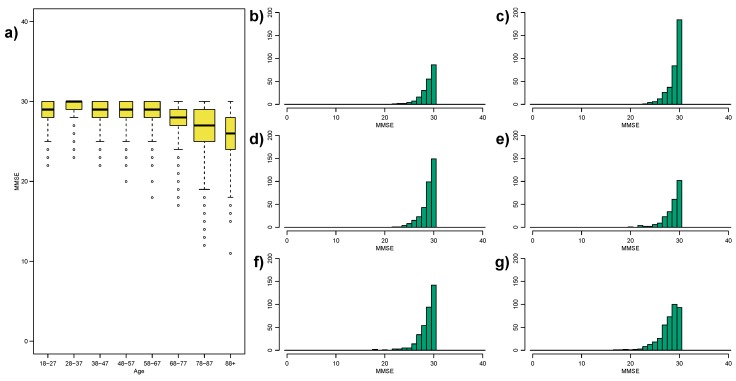
(**a**) Repetition of Figure 1c, note the extended y-axis to indicate the likely ceiling effect on the MMSE; (**b**–**g**) Histograms of the MMSE scores over the first six age-groups. The MMSE range is [0,30]. The 28–37 age-group (**c**) have a particularly large “spike” at the maximum score, indicative of a ceiling effect.

We can compare our simulated ceiling effect in Figure 5 to the MMSE ceiling effect in Figure 6. In our simulated ceiling effect we see “ceiling spikes” (the hatched bars in Figure 5b–g), where all individuals with scores above 33 have been recoded to 33. Conversely, the MMSE plots do not illustrate this type of “ceiling spike”–there are no clear peaks at an MMSE score of 30. However, in the MMSE case there may be some dilution of the “ceiling spike” as not everyone who encounters the ceiling effect will get an MMSE score of 30; we expect some of these “ceiling individuals” to score 29, or possibly even 28.

The MMSE is designed to detect cognitive variability that is clinically relevant (e.g., dementia risk) so it is not designed to be sensitive to individual differences within a normal/non-pathological range of cognitive ability. In particular, the 28–37 age-group appear to show the greatest spike-effect, which may reflect that age range having the best response to the MMSE measure.

In summary, despite the strong evidence against a constant variance for the MMSE and ACE-R scores (see Table 2), the ceiling effect is potentially violating the validity of Fligner’s test for equality of variances.

## 4. Conclusions

In this paper we have presented evidence and discussed several methodological issues in order to contribute to the debate for and against age-related increases in inter-subject variability [7,23]. We have shown that some commonly used cognitive measures actually exhibit stable variance across the adult lifespan, challenging a developing hypothesis from several ageing studies that variance increases in older age.

We posited that some reported results of increasing variability in older adults may be due to methodological issues: in Section 2.2 we consider the ill-defined concept of variability; in Section 3.3 we consider the increase in variance in an aggregated group actually being a divergence in location within sub-groups (of a possibly unmeasured confounder); and in Section 3.4 we consider how ceiling effects might impact the variance of younger age groups leading to an undefined comparison of biased group variances (the same issue would exist with a floor effect in older age groups).

There are several other methodological issues that may support or undermine our possible explanations. Firstly, it is well known that using inter-subject variability from cross-sectional data is not a substitute for longitudinal repeated observations; we have no way to assess the intra-subject variability over even a small time period without repeated observations. Secondly, the study may not accurately represent the population of interest; thus the observed changes in variance across the adult lifespan may be a result of biased sampling with age-groups.

With regard to the Cam-CAN study, the issue of using cross-sectional data as a substitute for observations across the adult lifespan remains. However the issue of biased sampling has been mitigated by the use of a population-based sample for the Cam-CAN study; whereas many cognitive studies utilise volunteer-cohort studies. The Cam-CAN study includes details of recruitment into each stage of the design [14], meaning our stage one sample is representative of the population (as can be seen by the wide education range represented).

As an aside, it is worth mentioning the issue of intra-individual variability which has recently become a popular measure of variability (see Hultsch *et al.* for a discussion of different types of variance [6]), e.g., using trial to trial, or session to session, variability as an explanation of increased inter-subject variability. Although this is linked to the debate for and against age-related increases in variability, it is a separate issue to our contribution.

We consider the box plot and two statistical tests, Mood and Fligner, as our methods to assess changing location and spread across the adult lifespan. As discussed in Section 2.4, the box plot is a sufficiently concise illustration to be useful in drawing comparisons across groups. The choice of statistical tests is not as clear cut. Although we consider non-parametric tests with minimal assumptions, whether these are the most statistically powerful in our situation is not definitive [19,24]. Among non-parametric tests, the Wilcoxon (or Mann-Whitney) test is commonly cited as a non-parametric equivalent of the t-test (the Kruskal-Wallis test being the generalisation to multiple groups). However, the Wilcoxon is not a test of equal medians unless you make assumptions on constant shape across groups, an assumption we cannot make in our setting. As indicated previously, the chosen statistical tests are not informative about the type of departure from equal means or variances, only that a departure has occurred.

We have shown that the delayed story recall exhibits constant variance across the adult lifespan, as a direct counter example to the claim that variability increases in cognitive measures for older adults, while the median delayed recall score declines with age [5]. However upon examining the delayed recall box plot in Figure 1b, we note that there appears to be a floor effect at the older ages, especially the 88+ age-group where the two whiskers are visually unlike the other age-groups. If we exclude this age group Fligner’s test gives a *p*-value of 0.174, concluding that the variance is constant across the other seven age-groups. The oldest age-group has two issues when comparing to the other seven, firstly as the smallest (with almost half as many individuals as other groups) and secondly it spans multiple deciles (age range is 88–102); we feel it is reasonable to conclude a constant variance across the adult lifespan for delayed recall. However, given the deficiencies of our 88+ group we cannot exclude that variability might increase among the very oldest adults. Both our measures of verbal fluency (category and letter) showed only weak evidence of changing variance, and we conclude that these fluency measures also exhibit a stable variance across the adult lifespan. The box plots, Figure 1e,f, show that the lower fluency scores are very close to the minimum score of zero; indicating a possible floor effect. However, unlike the delayed recall in Figure 1b, there appears to be no visual change in the whiskers across age-groups. Also unlike delayed recall, out of the 2680 individuals, only 4 had a zero score for letter fluency and there were no zeros for category fluency; compared to 15 zeros for story recall in the 88+ group and 38 in the 78–87 group. As discussed, we conclude there is no strong evidence against a constant variance for both fluency measures.

As a real, and quite dramatic, example of the problem of confounders affecting the spread as well as the location, the Wechsler immediate story recall initially showed a changing variance across the adult lifespan. However, when accounting for education attainment in Section 3.3 the result completely reversed, such that the variance within each education-group remained stable across the adult lifespan.

Finally we investigated the MMSE and ACE-R scores as common measures of general cognitive status. Both measures indicate increasing variance in older adults. However, upon inspection we see that both measures most likely suffer from a ceiling effect, making the comparison of variances across the age-groups potentially ill-defined. It might be said that the MMSE is deficient as a measure of global cognition across the adult lifespan due to its limited scale, (integer scores from 0 to 30), rather than a ceiling effect per se. The ACE-R score, of which the MMSE is a subset of the measure, has a finer scale (0–100) but still exhibits a ceiling effect for young adults, which may be driving the apparent age-related increase in variance. It is likely that, since the MMSE and ACE-R were designed as clinically-relevant scores, they may have issues with comparison across the adult lifespan. Future work will consider methods to detect and adjust for ceiling effects, or even floor and ceiling effects simultaneously, in variance comparisons; or if variance is the most appropriate measure of spread in the presence of truncation. An interesting question for future research is whether a test for truncation or even modified existing tests adjusting for truncation can be derived.

In summary, the Cam-CAN study is a large, population-based dataset with which we have considered the question of cognitive variability across the adult lifespan. We have shown that the question of variability depends on the specific measure, and that the simple calculation of changing variance without considering confounders or truncation may be giving an inaccurate impression of adult lifespan variability.
